# Phospho-regulation of ASCL1-mediated chromatin opening during cellular reprogramming

**DOI:** 10.1242/dev.204329

**Published:** 2024-12-12

**Authors:** Roberta Azzarelli, Sarah Gillen, Frances Connor, Jethro Lundie-Brown, Francesca Puletti, Rosalind Drummond, Ana Raffaelli, Anna Philpott

**Affiliations:** ^1^ Cambridge Stem Cell Institute, Jeffrey Cheah Biomedical Centre, Cambridge Biomedical Campus, Cambridge CB2 0AW, UK; ^2^ Department of Pharmacology, UCL School of Pharmacy, University College London, 29–39 Brunswick Square, London WC1N 1AX, UK; ^3^ Department of Oncology, University of Cambridge, Cambridge CB2 0XZ, UK

**Keywords:** Neurogenesis, Cell fate reprogramming, Proneural transcription factors, Embryonic stem cells, Chromatin accessibility, Phosphorylation

## Abstract

The proneural transcription factor ASCL1 regulates neurogenesis and drives somatic cell reprogramming into neurons. However, not all cell types can be reprogrammed by ASCL1, raising the questions of what provides competence and how we can overcome barriers to enable directed differentiation. Here, we investigate how levels of ASCL1 and its phosphorylation modulate its activity over progressive lineage restriction of mouse embryonic stem cells. We find that inhibition of ASCL1 phosphorylation enhances reprogramming of both mesodermal and neuroectodermal cells, while pluripotent cells remain refractory to ASCL1-directed neuronal differentiation. By performing RNA-seq and ATAC-seq in neuroectoderm, we find that un(der)phosphorylated ASCL1 causes increased chromatin accessibility at sites proximal to neuronal genes, accompanied by their increased expression. Combined analysis of protein stability and proneural function of phosphomutant and phosphomimetic ASCL1 reveals that protein stability plays only a marginal role in regulating activity, while changes in amino acid charge cannot fully explain enhanced activity of the serine-proline mutant variants of ASCL1. Our work provides new insights into proneural factor activity and regulation, and suggests ways to optimize reprogramming protocols in cancer and regenerative medicine.

## Teaser

**Highlighted Article:** The enhanced activity of phosphomutant ASCL1 depends on more efficient chromatin remodelling during *in vitro* neurogenesis.

## INTRODUCTION

The establishment of cell fate during embryonic development is orchestrated by transcription factors (TFs) that act alone or in combination to alter the chromatin landscape and patterns of gene expression. As cells commit to a specific lineage, they undergo significant changes in chromatin accessibility, alongside changes in their transcriptome, proteome and metabolic requirements. To achieve reprogramming, TFs must be able to read and eventually alter a chromatin landscape to establish lineage-specific domains of gene expression and impose epigenetic barriers that lock cell fate. ‘Master regulator’ TFs, often characterized by their pioneer activity, can open the chromatin at inaccessible or only partially accessible regions (Zaret and Carroll, 2011). These factors are typically expressed transiently, while activating a transcriptional cascade that sustains the lineage-specifying transcriptional programme over time.

The proneural basic helix-loop-helix (bHLH) TFs ASCL1 and NEUROG2 have been widely studied in brain development, in embryonic stem cells (ESCs) and in the field of cell fate reprogramming. ASCL1, in particular, can trans-differentiate fibroblasts and other somatic cell types, including astrocytes, hepatocytes and lymphoid cells, into neurons, often alongside co-factors (Heinrich et al., 2010; Karow et al., 2012; Pang et al., 2011; Tanabe et al., 2018; Vierbuchen et al., 2010; Wapinski et al., 2013). ASCL1 can also accelerate and enhance neuronal differentiation of cultured embryonic stem cells (Păun et al., 2023; Vainorius et al., 2023).

Recent work has shed further light on the distinct activity of ASCL1 and NEUROG2 in embryonic stem cells, where they drive different transcriptional programs (Aydin et al., 2019; Vainorius et al., 2023). Upon expression in ESCs, ASCL1 rapidly destabilizes the pluripotency network and drives neuronal differentiation. In contrast, NEUROG2-expressing cells pass through a neural stem cell intermediate stage that retains the pluripotency factor Sox2, reusing it to establish a neural stem cell program. During forced neuronal differentiation of ESCs by bHLH overexpression, ASCL1 and NEUROG2 activate both pan-neuronal and subtype specific neuronal programs, although the specific identity of the neurons generated *in vitro* does not fully recapitulate the types of neurons that these TFs would specify *in vivo* (Chanda et al., 2014; Vierbuchen et al., 2010; Zhang et al., 2013).

The E-box sequence recognized by bHLH TFs is generally conserved, but variations in the central two nucleotides can account for the largely distinct sets of sites bound by ASCL1 and NEUROG2 in ESCs and embryoid bodies (Aydin et al., 2019). Additionally, variations in the nucleotides adjacent to the canonical E-box also contribute to specificity, underscoring that the ability to bind to different sites lies mainly in the intrinsic DNA-binding properties of each transcription factor (Casey et al., 2018; Gordân et al., 2013).

The expression level of proneural bHLH proteins also influences their binding landscape. Inducing supraphysiological levels of ASCL1 levels results in TF recruitment to lower affinity sites that contain non-canonical and degenerate E-box motifs (Woods et al., 2022). Furthermore, post-translational modifications, including phosphorylation and ubiquitylation, regulate the timing and extent of pro-neural protein activity by integrating extra- and intracellular signalling information into the transcriptional program (Ali et al., 2020, 2014; Azzarelli et al., 2022; Gillotin et al., 2018).

Previously, we and others have shown that preventing phosphorylation of ASCL1 at critical serine residues enhances its ability to drive neuronal differentiation (Ali et al., 2020; Azzarelli et al., 2022; Ghazale et al., 2022; Li et al., 2014). This enhancement is generally attributed to increased protein stability and protein levels of dephosphorylated ASCL1, since phosphorylation can recruit ubiquitin ligases, leading to protein degradation (Sancho et al., 2014; Song et al., 1998). Similar mechanisms have been observed for the related factors NEUROG2 and NEUROG3 (Azzarelli et al., 2017; Manea et al., 2023; Sancho et al., 2014; Vosper et al., 2007), although this has not been directly tested for ASCL1.

In this study, we investigate how cellular context affects reprograming ability, by comparing the response of different ESC-derived lineages to forced ASCL1 expression and by dissecting the contribution of ASCL1 protein levels and phosphorylation status on its reprogramming activity. Our data indicate that mutating serine-proline phospho-acceptor sites to prevent phosphorylation increases ASCL1 protein stability, but, surprisingly, this is not the main driver of the enhanced activity. Instead, the intrinsic biochemical properties of the phospho-group and the specific amino acid in the serine-proline positions determine the activity of ASCL1. We have further shown that ASCL1 phosphorylation modulates its ability to increase chromatin accessibility at specific sites and that this chromatin remodelling results in distinct transcriptional changes. Taken together, our data point to the enhanced proneural activity of un(der)phosphorylated ASCL1 being dependent on its increased ability to open chromatin, and could reveal new ways of enhancing reprogramming in resistant and partially resistant contexts.

## RESULTS

### Competence to respond to ASCL1-mediated reprogramming differs in pluripotent, mesodermal and neuroectodermal environments

**Fig. 1.**
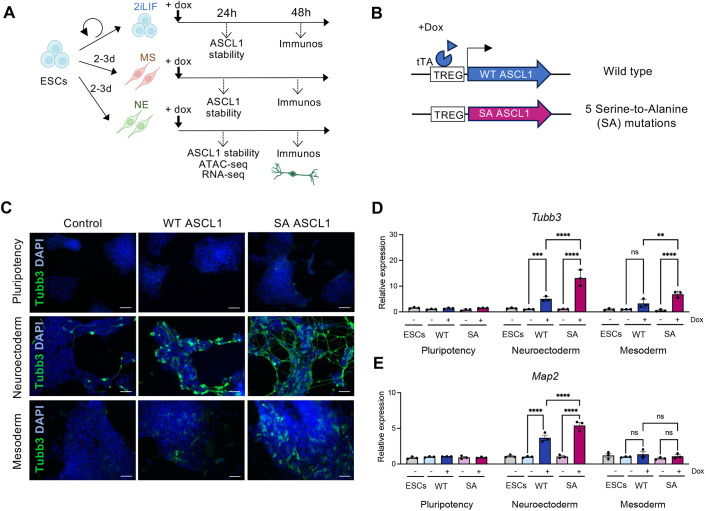
**Response to ASCL1 in pluripotency, mesoderm and neuroectoderm.** (A) Outline of the experiment: mouse embryonic stem cells (ESCs) were kept in pluripotency or driven down the neuroectoderm (NE) or mesoderm (MS) lineages before induction of wild-type (WT) ASCL1 or serine-to-alanine (SA) phosphomutant ASCL1. (B) Schematic of the lentiviral vectors carrying doxycycline (Dox)-inducible WT and SA ASCL1. (C) Representative immunostaining images for Tubb3 (green) in ESCs in pluripotency, NE and MS after 48 h of WT or SA ASCL1 induction or in control cells. DAPI nuclear counterstain (blue). Scale bars: 100 μm. (D,E) Relative mRNA expression of Tubb3 (D) and Map2 (E), normalised to β-actin, in the same conditions as described in C. Data are mean±s.e.m. (*n*=3 biological replicates), two-way ANOVA followed by Tukey's post-hoc test: **P<0.01, ***P<0.001, ****P<0.0001.

To uncover the mechanisms that control the ability of cells to respond to reprogramming factors, we initially tested the response of mouse embryonic stem cells (2iLIF) and ESC-derived mesodermal (MS) and neuroectodermal (NE) lineages to ectopic overexpression of the proneural transcription factor ASCL1 (human ASCL1 is used in mouse cells to discriminate endogenous and exogenous transcripts, while retaining high homology) (Fig. S1A-E for details of MS and NE differentiation). To test this, we generated ESCs carrying doxycycline-inducible ASCL1 (Fig. 1A,B; Fig. S1F). ASCL1 overexpression failed to induce Tubb3-positive neuroblasts in ESCs maintained in pluripotency media (Fig. 1C,D). Similarly, ESCs transferred to mesodermal media for 3 days also failed to show significant Tubb3 expression (Fig. 1C,D). In contrast, when ASCL1 was induced 3 days after transfer to neuroectodermal media, Tubb3-positive cells were frequently observed (Fig. 1C,D). Thus, despite its reported pioneer activity, ASCL1 cannot drive neurogenesis in pluripotent or mesodermal cells.

ASCL1 can be phosphorylated on multiple serine-proline (SP) sites. We have previously shown that phosphorylation on these sites limits the ability of ASCL1 to drive neuronal differentiation in some developmental and reprogramming settings (Ali et al., 2020, 2014; Azzarelli et al., 2022). To determine if preventing phosphorylation of ASCL1 enhances its ability to reprogramme pluripotent, neuroectodermal or mesodermal cells, we compared expression of Tubb3 in cells expressing wild-type (WT) ASCL1 or SA ASCL1, a mutant form in which all the serine-proline (SP) sites have been mutated to serine-alanine (SA) to prevent their phosphorylation (Fig. S1F). In NE media, SA ASCL1 was more active in driving neurogenesis than WT ASCL1, inducing greater expression of Tubb3 and also upregulating Map2, a marker of greater neuronal maturity (Fig. 1C-E). By contrast, in MS media, although phosphomutant ASCL1 was able to induce some Tubb3 expression, it could not upregulate Map2, indicating that barriers to further reprogramming still persist (Fig. 1C-E). Finally, when testing reprogramming ability in pluripotent conditions, we found that ESCs are unresponsive to both WT and SA ASCL1, as measured by Tubb3 or Map2 expression (Fig. 1C-E). These observations show that the reprogramming ability of ASCL1 is highly context dependent, and that this context dependency can only be partially overcome by changing bHLH factor phosphorylation status.

### Phosphorylation regulates ASCL1 protein stability in differentiated lineages

Our data show that some cell types are more responsive than others to ASCL1-mediated neurogenesis, and that phosphomutant ASCL1 is more effective than wild-type protein at driving Tubb3 expression in cells under neuroectoderm and mesoderm conditions. We next investigated what causes the enhanced reprogramming activity of phosphomutant ASCL1 in these systems. Previous studies on related bHLH factors NEUROG2 and NEUROG3 indicated that phosphorylation can regulate protein degradation (Azzarelli et al., 2017; Manea et al., 2023; Sancho et al., 2014; Vosper et al., 2007). However, little is known about the effect of phosphorylation on ASCL1 protein levels and stability.

**Fig. 2.**
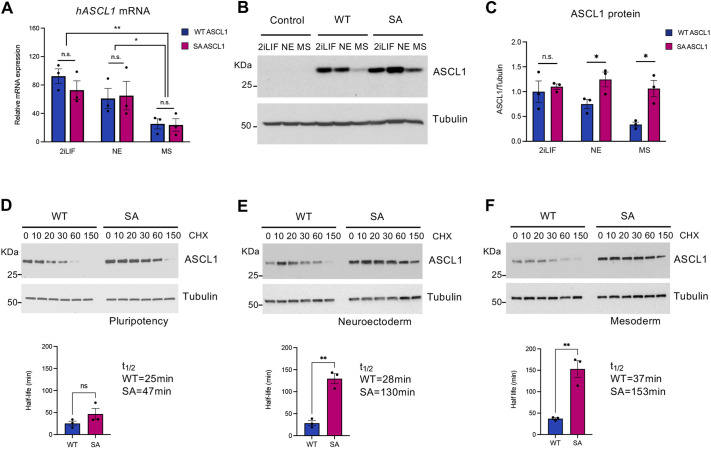
**Phosphorylation controls ASCL1 protein stability in a cell lineage-dependent manner.** (A) Quantification of ectopic *ASCL1* mRNA normalised to β-actin in ESCs in pluripotency (2iLIF), NE and MS after 24 h of wild-type (WT) or serine-to-alanine (SA) phosphomutant ASCL1 induction. An unpaired Student's *t*-test used to compare WT and SA in each media condition indicates no statistically significant differences (n.s.). A two-way ANOVA followed by Tukey's post-hoc test was used to test the effect of the media on ASCL1 expression (both WT and SA): **P=0.02 between 2iLIF and MS; *P=0.029 between NE and MS; no difference (*P*=0.32) between 2iLIF and NE (not shown on the graph). Data are mean±s.e.m. (*n*=3 biological replicates). (B) Representative western blot showing ASCL1 protein expression in control cells or after 24 h of WT and SA ASCL1 induction in the different media. (C) Quantification of ectopic ASCL1 protein relative to α-tubulin. Data are mean±s.e.m. (*n*=3 biological replicates). An unpaired Student's *t*-test used to compare WT and SA in each media condition: *P<0.05. (D-F) Representative western blot images used to calculate WT and SA ASCL1 protein stability in ESCs in pluripotency, neuroectoderm and mesoderm. Treatment time with cycloheximide (CHX) is in minutes. Graphs show the protein half-life (t_1/2_) of WT and SA ASCL1, calculated using first-order kinetics and simple linear regression. Data are mean±s.e.m. (*n*=3 biological replicates). An unpaired Student's *t*-test: **P<0.01.

We first examined ectopic *ASCL1* mRNA expression levels across pluripotent, MS and NE conditions. Surprisingly, significant differences in *ASCL1* mRNA levels were seen among the three conditions, with lower *ASCL1* levels in MS media (2iLIF versus MS, *P*=0.02; NE versus MS, *P*=0.029, two-way ANOVA followed by Tukey's post-hoc test) (Fig. 2A). While the reduced *ASCL1* mRNA levels in mesodermal conditions could contribute to relative resistance to reprogramming, differences in mRNA levels do not correlate with reprogramming ability, as cells in 2iLIF and NE exhibit similar levels of *ASCL1* mRNA, yet show a marked difference in reprogramming ability (Fig. 1). Furthermore, there are no significant differences in levels of WT compared to SA *ASCL1* mRNAs under each condition (Fig. 2A), indicating that the enhanced response to phosphomutant ASCL1 compared to WT is not due to higher SA mRNA levels. We also checked endogenous murine *Ascl1* expression across conditions and found the expected increase in NE cells that are going down the neuronal differentiation path, but no differences between WT and SA *Ascl1* in any of the conditions (Fig. S1G).

Next, we investigated the expression of ASCL1 protein across the different reprogramming conditions. In contrast to the mRNA levels, SA ASCL1 protein accumulates more in cells in NE and MS media compared to WT ASCL1 (Fig. 2B,C). We therefore investigated whether differences in ASCL1 protein stability could explain the differences in reprograming ability. Using cycloheximide to inhibit protein synthesis, we directly measured the half-life of ASCL1 in different media. WT ASCL1 exhibited a similar half-life across all conditions (ranging from 25±5 min in 2iLIF to 28±6 min in NE and 37±2 min in MS) (Fig. 2D-F). While there was a trend toward greater stability of SA ASCL1 in all conditions, this was only significant in cells in NE and MS conditions, where SA ASCL1 showed approximately a fourfold increase in stability compared to WT ASCL1 (130±11 min in NE; 153±19 min in MS) (Fig. 2D-F). Thus, SA ASCL1 shows increased protein stability in comparison to the WT, but this difference varies in the different environments.

### Protein level is not a key determinant of enhanced proneural activity of phosphomutant ASCL1

The enhanced reprogramming ability of phosphomutant ASCL1 in neuroectoderm and mesoderm cells could be attributed to its longer half-life compared to WT ASCL1. To investigate this, we adjusted doxycycline concentrations to achieve comparable protein expression of the WT and SA ASCL1, and tested their ability to induce Tubb3 expression in both NE and MS cells.

**Fig. 3.**
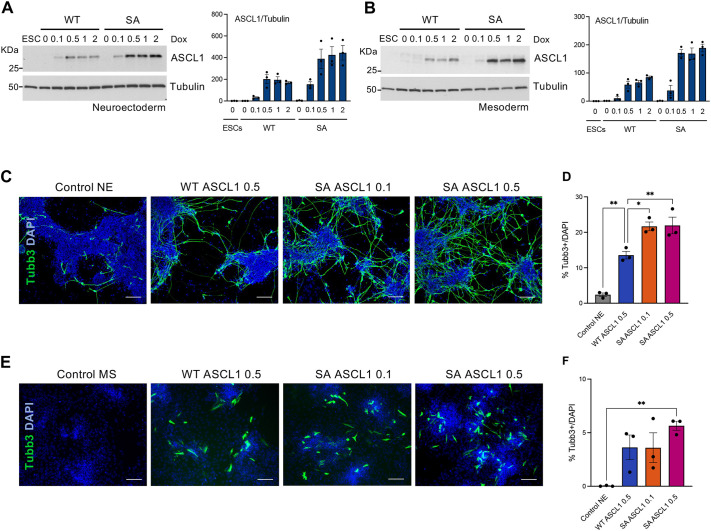
**Protein level contributes only marginally to enhanced pro-neural activity of phosphomutant ASCL1.** (A,B) Representative western blot images used to quantify wild-type (WT) or serine-to-alanine (SA) phosphomutant ASCL1 protein levels induced for 24 h by different doxycycline (Dox) concentrations, shown in µg/ml, in ESC-derived neuroectoderm (A) or mesoderm (B) lineages; untreated ESCs in pluripotency are shown as a control. Graphs show quantification of ectopic ASCL1 protein relative to α-tubulin. Data are mean±s.e.m. (*n*=3 biological replicates). (C,E) Representative immunostaining images for Tubb3 (green) in ESC-derived NE (C) and MS (E) after 48 h of WT or SA ASCL1 induction using either 0.1 or 0.5 µg/ml doxycycline or in control cells treated with 0.5 µg/ml of doxycycline. DAPI nuclear counterstain is in blue. Scale bars: 100 μm. (D,F) Quantification of Tubb3-positive cells compared to DAPI-positive cells. Data are mean±s.e.m. (*n*=3), from three independent biological experiments. One way ANOVA followed by Dunnett post-hoc test: *P<0.05, **P<0.01.

Comparable steady-state levels of WT and SA ASCL1 protein in NE cells were induced using 0.5 µg/ml and 0.1 µg/ml doxycycline, respectively (Fig. 3A). Despite these similar protein levels, phosphomutant SA ASCL1 was still significantly more neurogenic than WT ASCL1, as shown by Tubb3 expression (Fig. 3C,D). Moreover, increasing doxycycline concentration from 0.1 µg/ml to 0.5 µg/ml did not further enhance the Tubb3 expression in cells expressing SA ASCL1 (Fig. 3C,D). This finding demonstrates that the enhanced activity of phosphomutant ASCL1 in NE media cannot be fully explained by the increased protein levels and protein stability of SA ASCL1 compared to WT ASCL1. It is, however, important to note that the maximum reprogramming efficiency obtained in NE is only ∼25%. Tubb3-negative cells at 48 h could represent alternative lineages, as seen in other works on ESCs (Nishiyama et al., 2009; Vainorius et al., 2023), or it is possible that contributions from transgene silencing, insufficiently high levels of ASCL1 reached with the maximum doxycycline concentration and/or the short timing of induction might all influence the overall reprogramming efficiency.

We next investigated whether protein stability contributes to the enhanced reprogramming ability of SA ASCL1 in the less permissive MS environment. Similar to NE media, western blotting of cells in MS media showed that induction of WT ASCL1 and SA ASCL1 using 0.5 µg/ml and 0.1 µg/ml doxycycline, respectively, resulted in cells expressing comparative levels of steady-state ASCL1 protein (Fig. 3B). However, both WT and SA ASCL1, regardless of protein levels, exhibit low cellular reprogramming (∼4-5% Tubb 3-expressing cells in MS compared to 25% in NE) with a non-statistically significant trend towards more Tubb3-positive cells only in SA ASCL1 induced with 0.5 µg/ml doxycycline (Fig. 3E,F). This indicates that the MS environment is minimally responsive to ASCL1 expression and that the absolute amount of ASCL1 protein is not the limiting factor for neuronal reprogramming in this context.

Additionally, increasing doxycycline levels in pluripotent cells, to see whether induction of higher levels of WT or SA ASCL1 could overcome reprogramming barriers, yielded no Tubb3 expression (Fig. S2). The lack of response in pluripotent cells is likely due to the absence of necessary priming events (Rostovskaya et al., 2019). The mechanisms maintaining an undifferentiated state in the face of fate challenge in pluripotent cells may be distinct from those in lineage-committed cells.

Taken together, we have shown that the enhanced proneural activity of phosphomutant ASCL1 is not merely a consequence of increased protein levels. Instead, it is specific to the phosphorylation status of ASCL1. Given that pluripotent cells remain unresponsive to ASCL1-mediated neuronal differentiation and MS cells show minimal response despite changes in protein half-life, we aim to further investigate the mechanisms underlying the NE cell response to ectopic WT and SA ASCL1.

### Phosphorylation regulates the ability of ASCL1 to open chromatin in neuroectoderm

ASCL1 is a pioneer factor that can reprogram distantly related cell types because of its ability to open chromatin at inaccessible sites (Wapinski et al., 2017, 2013). However, the impact of ASCL1 phosphorylation on global chromatin remodelling is still poorly understood. To address this, we investigated how different levels of ASCL1 protein and its phosphorylation status influence chromatin accessibility using ATAC-seq after 24 h of induction with WT or phosphomutant SA ASCL1 in neuroectodermal cells. We analysed chromatin opening under the following conditions: WT ASCL1 induced using 0.5 µg/ml doxycycline and SA ASCL1 induced using either 0.5 µg/ml or 0.1 µg/ml doxycycline. These conditions allow us to differentiate the effect of protein levels from the effect of phosphorylation on chromatin opening, by comparing either WT 0.5 versus SA 0.1 (same protein levels, different phosphorylation status) or SA 0.1 versus SA 0.5 (different protein levels, same phosphorylation status) (Fig. S3A for PCA analysis).

**Fig. 4.**
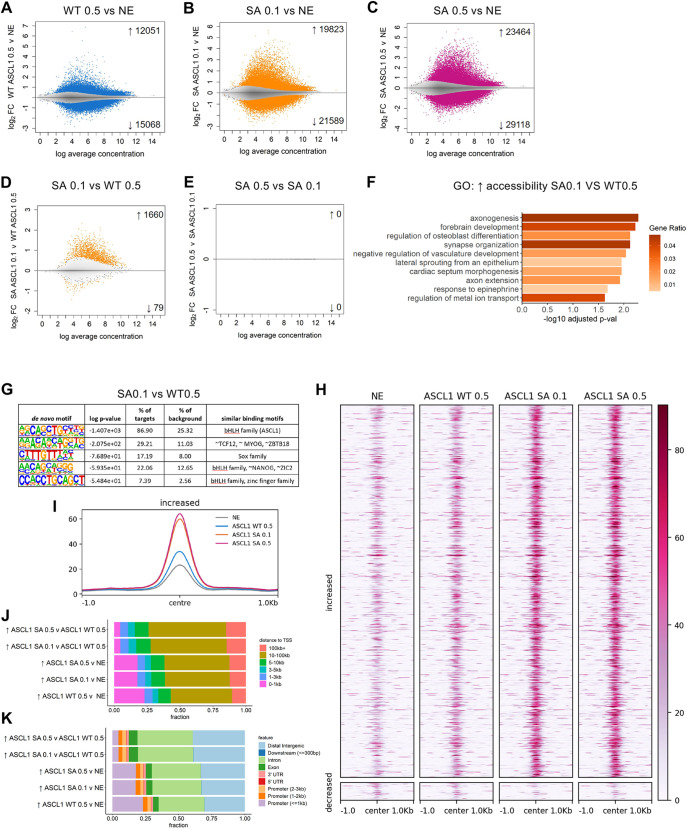
**Phosphomutant ASCL1 induces increased chromatin accessibility in neuroectoderm.** (A-E) Graphs showing differentially accessible regions (DARs) between conditions in ATAC-seq analysis. Wild-type (WT) and serine-to-alanine (SA) phosphomutant ASCL1 were induced by doxycycline treatment at 0.1 µg/ml (0.1) or 0.5 µg/ml (0.5) for 24 h. Neuroectodermal control (NE) samples were treated with 0.5 µg/ml doxycycline. (F) Gene ontology (GO) analysis for biological processes on the genes associated with increased accessibility in SA 0.1 versus WT 0.5. (G) Motif analysis comparing SA 0.1 versus WT 0.5. (H) Signal intensity ±1 kb around the peak summit of all regions with significantly altered accessibility in SA 0.1 versus WT 0.5, shown across conditions. (I) ATAC-seq data: normalised and averaged RPGC (reads per genomic content) over four replicates at sites with increased accessibly in SA 0.1 versus WT 0.5 are shown for all conditions. (J,K) Graphs showing that the increased DARs by SA ASCL1 are predominantly at distal regions rather than at promoters. Analyses were of four different biological replicates.

When comparing ATAC-seq signals in control neuroectodermal cells at day three of differentiation with cells overexpressing either WT or SA ASCL1, several thousands of genomic sites changed accessibility, indicating major widespread chromatin remodelling by both forms of ASCL1 (Fig. 4A-C). Notably, among the differentially accessible regions (DARs), SA ASCL1 induced a greater number of open regions compared to WT ASCL1 when protein levels were matched (1660 regions; Fig. 4D) and a more pronounced effect when comparing SA 0.5 µg/ml to WT 0.5 µg/ml (2902 regions; Fig. S3B). We did not see any significant DARs between cells expressing the different levels of SA ASCL1 protein (Fig. 4E). To investigate the genes impacted by the differentially accessibility, ATAC peaks were assigned to the most proximal gene. Genes associated with only increased accessibility sites in cells expressing SA ASCL1 at either level were strongly enriched for neuronal differentiation and axonogenesis terms compared to cells expressing WT ASCL1 (Fig. 4F, Fig. S3C; see Fig. S3D-F for GO terms in comparison to control NE).

Enrichment of Notch signalling components was seen in all comparisons, although it was amongst the top 10 terms only in WT ASCL1 0.5 versus control NE (Fig. S3D), overall indicating that both forms of ASCL1 maintain crosstalk with Notch signalling to regulate the balance between proliferation and differentiation.

We also looked at GO terms associated with the 79 (Fig. 4D) and 426 (Fig. S3B) peaks showing reduced chromatin accessibility in SA 0.1 versus WT 0.5 and in SA 0.5 versus WT 0.5, respectively. While the 79 genes were not enriched for any specific terms, the 426 genes provided only one term related to axon guidance, which might underlie regulation of subtype-specific maturation events. Overall, our ATAC-seq data show that the chromatin regions specifically opened by SA ASCL1 are strongly associated with neuronal genes, which might contribute to the enhanced neuronal differentiation phenotype.

We next asked whether the chromatin regions with increased accessibility upon overexpression of either WT or SA ASCL1, compared to control neuroectodermal cells, are enriched for specific transcription factor motifs. Motif discovery analysis revealed, as expected, an enrichment of the canonical E-box motif 5′-CAGCTG-3′ for ASCL1 (Aydin et al., 2019). This motif was present in 32% of WT 0.5 sites, in 51% of SA 0.1 sites and in 47% of SA 0.5 sites, with increased accessibility compared to NE (Fig. S4A-C). We also observed an enrichment of other motifs common to more accessible sites associated with both WT and SA ASCL1 compared to NE. These included motifs recognized by the transcriptional regulator CTCF, Sox proteins and POU domain-containing transcription factors. Notably, some motifs showed greater enrichment with WT or SA ASCL1 expression when compared to NE. For example, WT ASCL1 showed greater enrichment for motifs recognized by SP and KLF transcription factors. In regions more accessible in SA ASCL1-expressing cells compared to cells with similar levels of WT ASCL1 overexpression, we found that 87% of sites contain an ASCL1-specific E-box motif (Fig. 4G), suggesting these differentially accessible regions are directly regulated by ASCL1. Moreover, there was also an enrichment of motifs recognised by E proteins and other bHLH transcription factors, indicating that SA ASCL1 has a particularly strong preference in remodelling E-box-containing regions. However, our analysis did not reveal any preference for different consensus sequences between WT and SA, suggesting that WT and phosphomutant ASCL1 can potentially bind to the same sites. Indeed, when we mapped the regions with greater accessibility in cells overexpressing SA ASCL1 0.1 compared to WT ASCL1 across all conditions, we found that these regions all exhibit a low signal in the WT ASCL1-expressing cells, though this difference was not statistically significant (Fig. 4H,I). It is therefore possible that WT and SA ASCL1 might share similar binding sites, but phosphomutant ASCL1 is better able to remodel the chromatin at those sites. Interestingly, when we looked at the location of these DARs that are more accessible in SA versus WT, we found a shift from promoter regions (0-1 kb) to more distal regions (10-100 kb), either distal intergenic or intronic (Fig. 4J,K), suggestive of increased chromatin remodelling by phosphomutant ASCL1 to potential enhancers compared to the wild-type protein. Overall, we have shown that preventing ASCL1 phosphorylation increases chromatin remodelling in regions associated with neuronal genes in ESC-derived neuroectoderm and particularly at potential enhancers. This suggests that the phosphorylation status of ASCL1 might regulate its effectiveness as a pioneer factor or chromatin remodeller at key regulatory regions.

### Impact of phosphorylation of ASCL1 on genome-wide transcription in neuroectoderm

**Fig. 5.**
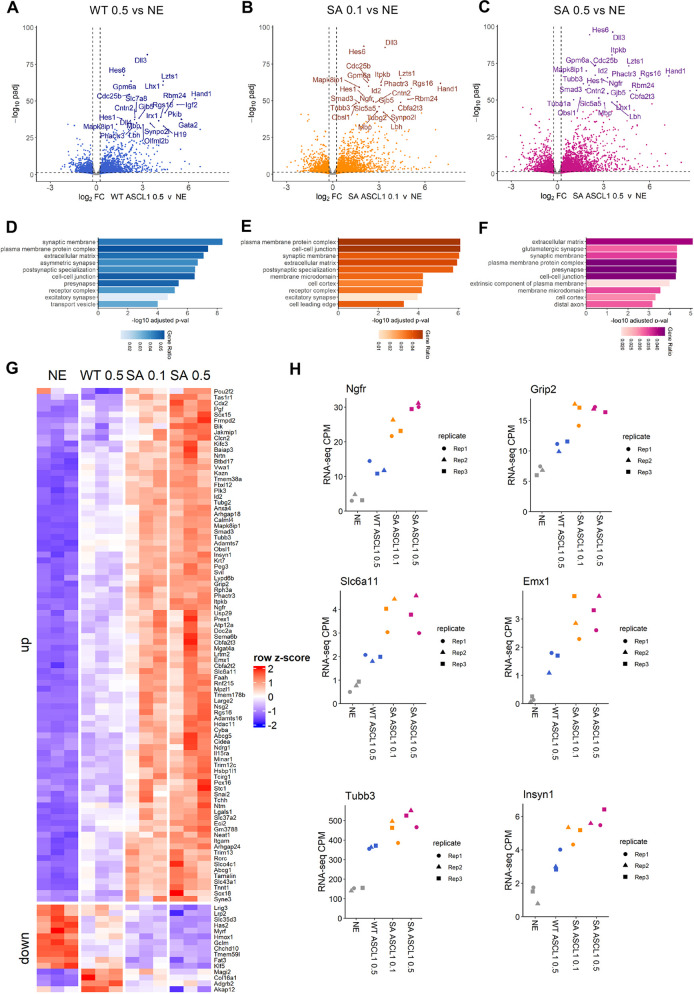
**Transcriptomic changes mediated by phosphomutant ASCL1 in neuroectoderm.** (A-C) Volcano plots of differential RNA-seq changes. Wild-type (WT) and serine-to-alanine (SA) phosphomutant ASCL1 were induced by doxycycline treatment at 0.1 µg/ml (0.1) or 0.5 µg/ml (0.5) for 24 h. Neuroectodermal control (NE) samples were treated with 0.5 µg/ml doxycycline. Differential changes are shown for (A) WT 0.5 versus NE, (B) SA 0.1 versus NE and (C) SA 0.5 versus NE. Highlighted are the top 25 significant differentially expressed genes (*P*.adj<0.05 and log2FC> 0.25<−0.25). (D-F) Gene ontology analysis for cellular components of upregulated genes in the comparisons shown in A-C, respectively. (G) Heatmap highlighting differentially expressed genes between SA 0.5 versus WT 0.5, shown across conditions. The expression of these genes (normalised counts per million and z-score scale across the samples) is shown across the conditions: NE control, WT 0.5, SA 0.1 and SA 0.5 for three biological replicates. (H) RNA-seq expression (counts per million, CPM) shown for specific individual genes. Data points are for the three biological replicates.

Given that ASCL1 phosphorylation strongly impacts the accessibility of the chromatin in neuroectoderm, we next investigated whether this chromatin remodelling results in transcriptional changes underlying enhanced neuronal differentiation. We mirrored conditions for our ATAC-seq data, carrying out RNA sequencing of neuroectodermal cells 24 h after induction of WT or SA ASCL1. Overexpression of either WT ASCL1 or phosphomutant ASCL1 leads to genome-wide transcriptomic changes when compared to control ESC-derived neuroectoderm (Fig. 5A-C; Fig. S5A,B).

Genes upregulated by either WT or SA ASCL1 were associated with gene ontology terms including synaptic membrane, postsynaptic specialization and excitatory synapses (Fig. 5D-F), suggesting that both forms of ASCL1 can cause enhanced neuronal differentiation. However, while both WT or SA ASCL1 induction resulted in expression changes for over three thousand genes, few genes were significantly differentially expressed between the two, regardless of whether the level of SA and WT ASCL1 proteins were matched. Specifically, 28 genes were upregulated and two genes downregulated by SA 0.1 compared to WT 0.5, and 88 genes were upregulated and 15 genes downregulated by SA 0.5 compared to WT 0.5 (Fig. S5A). Only one gene was differentially expressed between cells expressing the different levels of phosphomutant ASCL1.

**Fig. 6.**
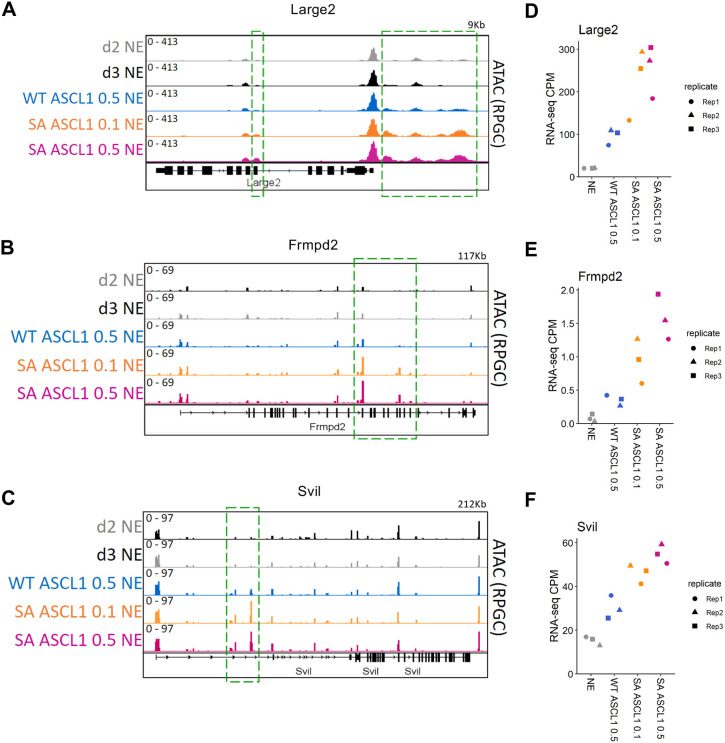
**Correlation between increased genome accessibility and transcription mediated by phosphomutant ASCL1 in neuroectoderm.** (A-F) Genome browser tracks of accessibility surrounding three example genes that exhibit both association with chromatin regions of increased accessibility (A-C) and significant upregulation at the RNA level (D-F) in serine-to-alanine (SA) phosphomutant ASCL1 versus wild-type (WT) ASCL1 conditions. WT ASCL1 and SA ASCL1 were induced by doxycycline treatment at 0.1 µg/ml (0.1) or 0.5 µg/ml (0.5) for 24 h. Control samples were in NE differentiation media for 2 or 3 days (d2 NE and d3 NE). For each condition, the tracks show the average of four biological replicates with reads per genomic content normalisation (RPGC). Green dotted lines highlight the regions that are significantly different between SA ASCL1 0.5 and WT ASCL1 0.5. Region size (in kb) is indicated in top right of each plot. (D-F) RNA-seq expression level (counts per million) for the three example genes: (D) Large2, (E) Frmpd2 and (F) Svil.

Next, we interrogated the genes differentially expressed between WT 0.5 and SA 0.5 by comparing expression across all conditions (Fig. 5G). Among the 88 genes upregulated by SA 0.5 in comparison to WT ASCL1, approximately one-third were also significantly upregulated by SA 0.1 (28 genes) and the remaining genes exhibit a trend towards upregulation in SA 0.1, despite not reaching significance (Fig. 5G). Most of these genes are involved in neuronal differentiation; examples include the nerve growth factor receptor Ngfr, the glutamate interacting protein Grip2 and the transporter responsible for gamma-aminobutyric acid (GABA) uptake Slc6a11 (Fig. 5H). Interestingly, integration of RNA-seq and ATAC-seq data revealed that more than 65% of the 88 genes upregulated by SA 0.5 compared to WT ASCL1 also showed increased chromatin accessibility in their proximity (Fig. S5C) (examples of Large2, Frmdp2 and Svil are shown in Fig. 6). These data suggest that, 24 h after induction, the phospho-status of ASCL1 has only a small impact on its ability to drive gene expression in neuroectoderm, despite the increased chromatin accessibility. It is, however, worth mentioning that bulk analysis of a cell population where only ∼25% of the cells are reprogrammed (Fig. 3C,D) might in fact limit the magnitude of the differences we see between WT and SA ASCL1.

In summary, we have shown that phosphorylation of ASCL1 plays a major role in controlling accessibility and opening of the chromatin. However, the subsequent changes in gene expression leading to enhanced neuronal differentiation might be due to a cascade of events triggered by this enhanced chromatin accessibility.

### ASCL1 phospho-mimetic does not show reduced proneural activity

Phosphorylation of ASCL1 can enhance its protein stability and ability to drive neuronal reprogramming. However, even when matched for protein levels, phosphomutant ASCL1 is more effective than WT in inducing neurogenesis in neuroectoderm. We sought to probe further the additional properties of SA ASCL1 that mediate its increased proneural activity compared to the wild-type protein.

**Fig. 7.**
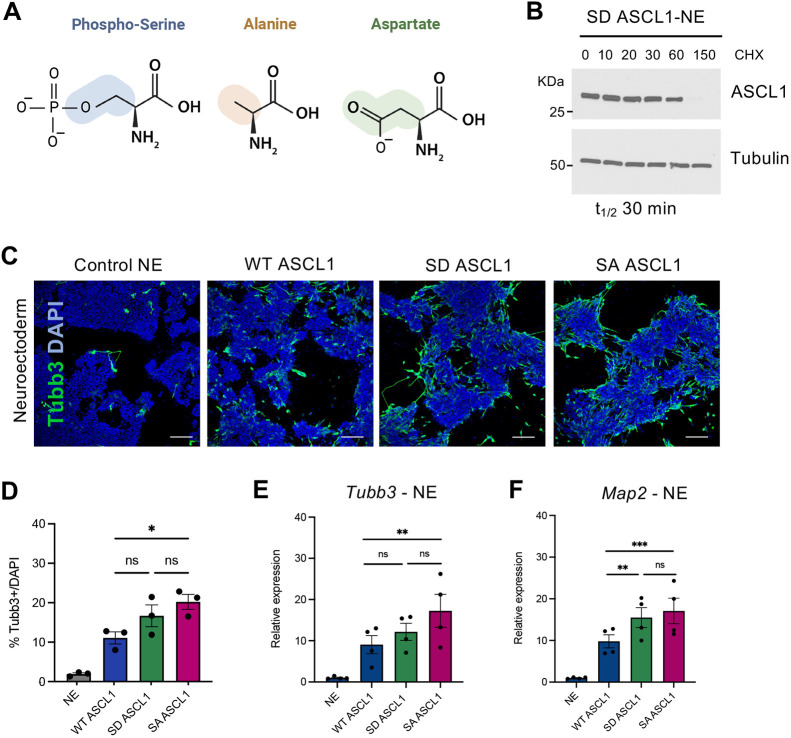
**ASCL1 phospho-mimetic does not show reduced proneural activity.** (A) Schematic showing the structure and main properties of serine, alanine and aspartic acid. (B) Representative western blot used to calculate SD ASCL1 protein stability in ESC-derived neuroectoderm (NE). Treatment time with cycloheximide (CHX) is indicated in minutes. Protein half-life (t_1/2_) is the mean of three biologically independent experiments, calculated using first-order kinetics and simple linear regression. (C) Representative immunostaining images for Tubb3 (green) in ESC-derived neuroectoderm after 48 h induction of wild-type (WT), SD or serine-to-alanine (SA) phosphomutant ASCL1 using 0.5 µg/ml doxycycline or in doxycycline-treated control neuroectodermal cells. DAPI nuclear counterstain (blue). Scale bars: 100 μm. (D) Quantification of Tubb3-positive cells compared to DAPI-positive cells in neuroectoderm. Data are mean±s.e.m. (*n*=3 biological replicates). One way ANOVA followed by Tukey's post-hoc test: *P<0.05. Statistical differences against control NE are not shown (NE versus WT *P*=0.03; NE versus SD *P*=0.002; NE versus SA *P*=0.0005). (E,F) Relative mRNA expression of neuronal markers *Tubb3* (E) and *Map2* (F), normalised to β-actin, in neuroectoderm (NE) after 48 h induction of WT, SD and SA ASCL1. Data are mean±s.e.m. (*n*=3 biological replicates). One way ANOVA followed by Tukey's post-hoc test: *P<0.05, **P<0.01, ***P<0.001. Statistical differences against control NE not shown (*Tubb3*: NE versus WT n.s. *P*=0.16; NE versus SD *P*=0.037; NE versus SA *P*=0.03; *Map2*: NE versus WT n.s. *P*=0.053; NE versus SD *P*=0.018; NE versus SA *P*=0.0008).

Proline-directed serine phosphorylation of ASCL1 introduces negatively charged, hydrophilic groups into the protein structure. To test the specific contribution of these negative charges to controlling ASCL1 activity, we generated a phospho-mimetic version of ASCL1 by mutating the serine residues into aspartic acid at all five of the available SP sites (referred to as the SD mutant). This SD mutant mimics phosphorylated ASCL1 in that it carries constitutive negative charges at the same locations as a fully phosphorylated protein (Fig. 7A; Fig. S6A-C). We hypothesised that mimicking constitutive phosphorylation of ASCL1 would result in decreased protein stability and reduced differentiation capability in comparison to WT ASCL1. However, we found that SD ASCL1 protein had a half-life of 30±4 min in neuroectoderm (Fig. 7B) and 27±4 min in mesoderm (Fig. S6D), which is similar to the stability of WT ASCL1 (Fig. 2E,F). Moreover, when we induced SD ASCL1 expression in NE, we found that it was not minimally active, but instead it promoted neurogenesis at least as well as WT ASCL1 (Fig. 7C-F). There was indeed a trend for SD ASCL1 activity to be slightly greater than WT ASCL1, which reached significance when measuring the expression of the neuronal gene *Map2* (Fig. 7F). In addition, when compared to the activity of SA ASCL1, SD activity was never statistically significantly different, indicating that SD ASCL1 might have an intermediate level of activity between WT and SA ASCL1 (Fig. 7C-F). Similar results were observed in mesodermal conditions, where SD ASCL1 also exhibits a level of activity not statistically significantly different from either WT or SA ASCL1 (Fig. S6E,F). These surprising results reveal that the mechanism underlying phospho-regulation of ASCL1 does not rely solely on the addition of negative charges, but likely on other biochemical properties of the amino acid in the serine position at the SP sites.

## DISCUSSION

ASCL1 is a master regulator of neurogenesis with a pivotal role in the development of the central and peripheral nervous systems. We have previously shown that phosphorylation of ASCL1 on multiple serine-proline sites regulates its ability to drive neuronal differentiation in the developing embryo (Ali et al., 2014). ASCL1 has also emerged as a key factor that can be used to convert cells from an alternative lineage into a neuronal fate (Chanda et al., 2014; Dennis et al., 2019; Wapinski et al., 2013). However, the efficiency of reprogramming using ASCL1 varies considerably between cell types, and manipulating its post-translational modifications might offer a way to enhance this reprogramming process.

In this study, we explored ASCL1 programming activity in embryonic stem cells and ESC-derived partially defined lineages. By comparing the effect of mimicking or preventing ASCL1 phosphorylation, we aimed to gain mechanistic insights into how these modifications modulate the ability of ASCL1 to drive neuronal programming. Previous studies have indicated a complex role for post-translational phosphorylation of ASCL1. For example, in cancer cells, unphosphorylated ASCL1 can increase neuronal differentiation and contribute to reducing tumorigenesis by forcing cells to exit the cell cycle (Ali et al., 2020; Azzarelli et al., 2022). However, in other contexts, phosphorylation by ERK biases ASCL1 in favour of driving a glial differentiation programme (Li et al., 2014). Additionally, in oligodendrocyte progenitor cells, ASCL1 overexpression results in increased proliferation rather than driving cells to differentiate (Galante et al., 2022), although the role of phosphorylation in this context is still unknown.

We investigated whether ASCL1 de-phosphorylation could overcome the barriers to neuronal differentiation in pluripotent cells and enhance the efficiency of reprogramming in ESC-derived neuroectodermal and mesodermal cells. We found that, while overexpression of unphosphorylated ASCL1 can enhance differentiation and reprogramming in NE and MS, it could not drive a neuronal programme in cells maintained in pluripotency conditions. The reasons behind pluripotent cell resistance to ASCL1-mediated reprogramming are not clear, and integrated analysis of chromatin accessibility, ASCL1 binding and presence/absence of co-factors would be essential to define the main contributors to ESC resistance.

The enrichment of Sox motifs in ASCL1 peaks in NE might indicate that interaction with Sox TFs or recruitment of Sox TFs to ASCL1-binding sites could be significant for neuronal differentiation. However, Sox2, which enhances ASCL1-mediated neuronal reprogramming in other contexts (Karow et al., 2018, 2012) is highly expressed in ESCs and yet ESCs do not respond to ectopic ASCL1 by undergoing neurogenic differentiation. Thus, it is likely that a combination of factors is involved in shaping the response to ASCL1.

It is also possible that alternative lineages are activated by ASCL1 in ESCs and mesoderm. ASCL1 is known to activate skeletal muscle and cardiomyocyte genes in fibroblasts (Treutlein et al., 2016) and can enhance cardiac reprogramming when co-expressed with the TF Mef2c (Wang et al., 2022). Thus, future bulk and single cell transcriptomic analyses are needed to address the role of ASCL1 in driving alternative lineages from ESCs and MS.

Post-translational modifications often have significant effects on protein half-lives (Azzarelli et al., 2017; Manea et al., 2023; Sancho et al., 2014; Song et al., 1998; Vosper et al., 2007). Here, we show that phosphorylation accelerates the degradation of ASCL1 protein, whereas un(der)phosphorylated ASCL1 protein accumulated due to its increased stability. Specifically, phosphomutant ASCL1 exhibits a fourfold increase in stability compared to WT ASCL1 in NE and MS cells, but only a 1.8-fold increase in pluripotent cells. This differential regulation suggests that ASCL1 stability in pluripotent cells may be regulated differently, possibly via ubiquitin ligases other than Huwe1, which has been shown to regulate ASCL1 protein dynamics in neural stem cells (Gillotin et al., 2018; Urbán et al., 2016).

Neuroectoderm and mesoderm lineage-restricted cells accumulate higher levels of SA ASCL1 protein compared to cells expressing WT ASCL1. We reasoned that this could account for the increased proneural activity of the unphosphorylated protein in these contexts. However, even when WT and SA ASCL1 were matched for protein levels, SA ASCL1 was more neurogenic, especially in neuroectoderm. This shows that there is no direct correlation between phosphorylation, protein stability/steady-state levels and proneural activity. Our findings are consistent with previous work looking at ASCL1 phosphorylation on serine S152 (Viñals et al., 2004): mutations that prevent S152 phosphorylation resulted in a less stable but more active ASCL1. Hence, we cannot infer activity of ASCL1 solely based on protein levels or changes in the phosphorylation of individual residues, but instead must look to more nuanced mechanisms of post-translational control. It is likely that there are multi-layered controls of ASCL1 expression and post-translational modifications that provide ways to refine its proneural activity.

**Fig. 8.**
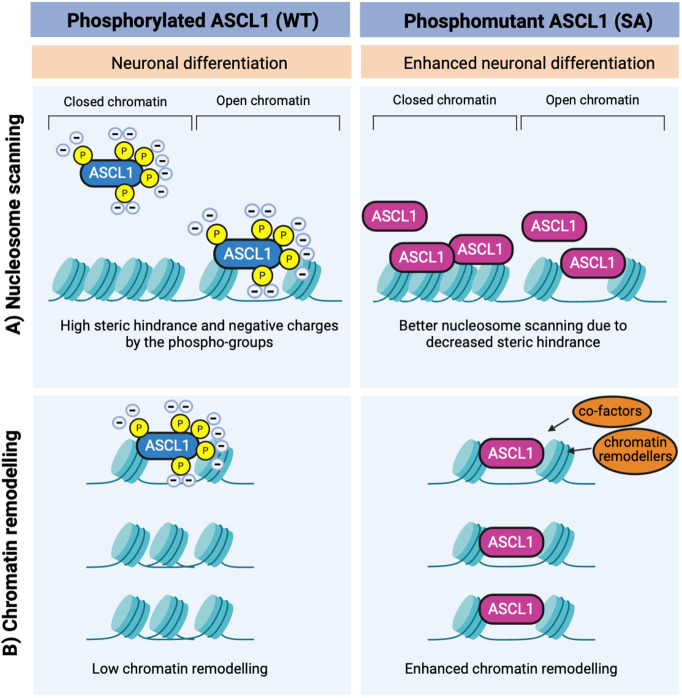
**Models of ASCL1 phospho-regulation in ESC-derived neurogenesis.** (A,B) The enhanced chromatin accessibility upon serine-to-alanine (SA) phosphomutant ASCL1 expression in comparison to wild-type (WT) ASCL1 can result from increased SA ASCL1 ability to (A) scan the nucleosome and/or (B) open the chromatin via co-factor recruitment. Created in BioRender. Lundie-Brown, J. (2024) https://BioRender.com/v28i792.

Our data led us to hypothesise that preventing phosphorylation of ASCL1 has an effect on its activity that goes beyond increasing protein levels and likely lies in the intrinsic properties of the phosphomutant protein, possibly due to changes in protein charge. We therefore tested the activity of a phosphomimetic version of ASCL1. This serine-aspartate mutant (SD) mimics phosphorylation at the serine-proline sites in that it brings a constitutive presence of negative charges. Surprisingly, the activity of SD ASCL1 mutant was not reduced in comparison to that of WT ASCL1. However, the aspartates that are substituted for the serines would mimic only one of the two negative charges of a phosphate group and might lack the steric hindrance carried by the phosphate groups (Fig. 7A). Thus, in addition to a constitutive negative charge, partial reduction in negative charges and in steric hindrance might underlie the retained activity of SD ASCL1. Altogether, these results point to a minor contribution of the negative charges to the enhanced phenotype of phosphomutant SA ASCL1 and indicate instead that other properties related to the type of amino acid in the position of the phospho-serines in SP sites are responsible for modulating ASCL1 activity. Phosphorylation could also influence the electrostatic interactions that control the capacity of ASCL1 to form condensates (Sridharan et al., 2022; Valverde et al., 2023) and/or might impose a steric hindrance that influences its ability to scan the nucleosome and interact with DNA (Fig. 8). Indeed, our data show that phosphorylation of ASCL1 restricts the opening and remodelling of the chromatin at neuronal loci, which is consistent with the recently published work on the related proneural bHLH NEUROG2 (Pereira et al., 2024), while additional mechanisms may control enhanced reprogramming downstream of increased chromatin accessibility. It is possible that phosphomutant ASCL1 is intrinsically better at scanning the nucleosome at regions that are only partially permissive or closed (Fig. 8A) and/or it can more effectively remodel the chromatin by recruiting chromatin remodellers, such as members of the SWI/SNF complex (Fig. 8B) (Janas et al., 2022; Păun et al., 2023).

In summary, the proneural activity of the pioneer transcription factor ASCL1 can be modulated by phosphorylation. In this study, we have shown that un(der)phosphorylated ASCL1 increases chromatin opening at sites proximal to neural genes. Mechanistically, we have evidence that the main driver of this enhanced activity of unphosphorylated ASCL1 lies in the intrinsic properties of the serine residues at SP sites, rather than in increased protein stability. However, cellular context can strongly influence the response to ASCL1, regardless of its phosphorylation status, suggesting that this post-translational modification is part of a complex regulatory environment. Defining the players in this regulatory landscape will provide novel tools to target the activity of ASCL1 and of other related transcription factors to improve differentiation and reprogramming in diseases.

## MATERIALS AND METHODS

### Embryonic stem cell culture

The mouse embryonic stem cell line E14 was kindly gifted by Prof. Jennifer Nichols, University of Edinburgh, UK (Mulas et al., 2019; Ying et al., 2008). Cells were cultured on 0.1% gelatin-coated plates in N2B27 medium supplemented with 2iLIF. The composition of N2B27 media was a 1:1 mixture of Dulbecco's Modified Eagle's Medium:Ham's F-12 media (DMEM:F12; Gibco, D6421) and Neurobasal medium (Gibco, 21103049), supplemented with 0.5× B27 (Gibco, 17504044), 1× N2 (in-house), 1× penicillin/streptomycin (Sigma, P0781), 2 mM L-glutamine (Gibco, 25030024) and 50 μM 2-mercaptoethanol (Thermo Scientific, 125472500). N2B27 was supplemented with 100 U/ml mouse leukaemia inhibitory factor (LIF, in-house, Cambridge Biochemical Department), 1 μM PD0325901 (ABCR, AB253775) and 3 μM CHIR 99021 (ABCR, AB253776). Cells were routinely passaged every other day. Cells tested negative for *Mycoplasma*.

### Differentiation of embryonic stem cells

#### Neuroectoderm differentiation

To differentiate ESCs towards the neural lineage, we followed a previously described protocol (Mulas et al., 2019; Ying et al., 2003). ESCs were cultured on plates coated with 10 μg/ml laminin I (R&D Systems, 3446-005-01). Neural differentiation medium was composed of N2B27 medium supplemented with 0.1% sodium bicarbonate (Sigma, S8761), 0.11% Bovine Serum Albumin Fraction V (BSA; Gibco, 15260037) and 20 μg/ml insulin (Sigma, I9278).

#### Mesoderm differentiation

To differentiate ESCs towards the presomitic mesodermal lineage, we followed a previously described myogenic protocol (Chal et al., 2018). ESCs were cultured on 0.1% gelatin-coated plates in N2B27 medium supplemented with 1% Knock-out Serum Replacement (KSR; Gibco, 10828010), 25 μg/ml BSA (Gibco 15260037) and 10 μg/ml insulin (Gibco, 12585014) for 2 days. On day two, medium was changed to DMEM high glucose (Gibco, 41965039) with 15% fetal bovine serum (FBS; PAN-Biotech, P40-37500), 0.1 mM 2-mercaptoethanol (Thermo Scientific, 125472500), 5 μM CHIR 99021 (ABCR, AB253776), 0.5% dimethyl sulfoxide (DMSO; ChemCruz SC-358801) and 0.1 μM LDN193189 (Tocris, 6053) for 2 days. On day four, medium was changed to a reduced-serum medium: DMEM high glucose containing 1% FBS, 14% KSR, 0.1 mM 2-mercaptoethanol, 5 μM CHIR 99021, 0.5% DMSO and 0.1 μM LDN1931189.

### Plasmid generation and mutations of ASCL1

#### Generation of phosphomutant (SA) and phosphomimetic (SD) ASCL1

The serines of the serine–proline sites in position 93, 190, 194, 207 and 223 of the human ASCL1 cDNA were mutated into alanine-proline (previously shown in Ali et al., 2020, 2014; Azzarelli et al., 2022) and into aspartate-proline. To mutate serines into aspartate, two rounds of amplifications with the QuikChange Multi Site-Directed Mutagenesis kit (Agilent Technologies, 200515) were performed (primers are in Table S1).

#### Generation of lentiviral vectors

WT, SA and SD ASCL1 cDNAs were cloned into pLVX-TRE3G (Clontech) between BamHI and EcoRI sites. SD ASCL1 contains an N-terminal HA tag.

### Lentiviral infection to generate ESCs expressing inducible ASCL1

The day after plating in N2B27-2iLIF media, ES cells were infected with two lentiviral vectors: (1) pLVX-Tet3G vector (Clontech) and (2) pLVX-TRE3G vector (Clontech) containing either WT ASCL1, SA ASCL1 or SD ASCL1 under the Tet-responsive promoter. Double selection was carried out using 1 μg/ml puromycin (Gibco, A1113803) and 300 μg/ml geneticin G418 (Sigma, G8168). Resistant cells were expanded as pools. Transgenes were induced using doxycycline (Clontech, 631311).

### PCR

RNA was extracted using the RNeasy mini kit (Qiagen, 74106) and reverse transcribed to cDNA using the QuantiTect Reverse Transcription kit (Qiagen, 205314). Quantitative PCRs were performed using PowerUp SYBR Green Master Mix (Applied Biosystems, A25742) and run either on the ViiA 7 Real-Time PCR System with 384-well block (Applied Biosystems) or on the StepOne Plus with 96-well block (Applied Biosystems). A list of primers used in the qPCR reactions can be found in Table S2. Data were analysed using the Delta Delta Ct formula (Livak and Schmittgen, 2001) and statistical significance was calculated using either one-way or two-way ANOVA, depending on the number of samples and type of comparisons. Details of the statistical test used are described in the figure legends.

### Immunocytochemistry

Cells were fixed with 4% paraformaldehyde (ThermoFisher Scientific, J19943-K2) for 15 min at room temperature. Cells were permeabilized with 0.2% Triton X-100 (Sigma, X100) in PBS for 15 min and blocked with 0.01% Triton X100-PBS containing 5% serum for 45 min at room temperature. Cells were incubated overnight at 4°C with the primary antibody, mouse anti-TUBB3 (1:2000; Biolegend, 801201- RRID:AB_2313773) in 0.01% Triton X100-PBS containing 2% serum. The next day, cells were washed using PBS and incubated for 2 h at room temperature with the secondary antibody, Alexa Fluor 488 anti-mouse IgG (Invitrogen, A21202) diluted 1:800 in 0.01% Triton X100-PBS containing 2% serum. Cells were stained with 1 μg/ml DAPI staining solution (Abcam, ab228549) for 20 min at room temperature.

### Imaging and image analysis

Fluorescent images were taken using the SP5 or Stellaris confocal microscopes (Leica) or the DMI4000 inverted microscope (Leica). CellProfiler v.4.2.1. (Stirling et al., 2021) was used to quantify Tubb3-positive cells using a minimum of three images from three biologically independent experiments (e.g. cells plated, differentiated and induced in separate experiments). Quantification of Tubb3-positive cells between control, WT ASCL1, SA ASCL1 and SD ASCL1 is shown as mean±s.e.m., calculated using CellProfiler. Statistical differences were analysed using one-way ANOVA. Details of the statistical test used are described in the figure legends.

### Immunoblot analysis

Cell lysates for western blots were collected in RIPA buffer (Sigma, R0278) supplemented with protease inhibitors (Sigma, 4693159001) and phosphatase inhibitors (Sigma, 4906845001). Protein quantification was performed using the Pierce BCA protein assay kit (Thermo Scientific, 23227). Proteins were separated on SDS PAGE 4-20% gradient gels (Bio-Rad, 5671094) and transferred onto nitrocellulose membrane (Bio-Rad, 1620115). Membranes were blocked in 5% milk (Serva 42590.02) in TBST [TBS containing 0.1% Tween (Promega, H5151)] and then incubated overnight at 4°C with primary antibodies: rabbit anti-ASCL1 (1:1000; Abcam, ab211327- RRID:AB_2924270), mouse anti- α-Tubulin (1:10,000; Proteintech 66031-1-Ig-RRID:AB_11042766) and/or mouse anti-GAPDH (1:2000; ThermoFisher, ma5-15738-RRID:AB_10977387). After washing in TBST, membranes were incubated with secondary antibodies, anti-rabbit IgG HRP-conjugated (1:10,000; Cytiva NA934- RRID:AB_772206) and anti-mouse IgG HRP-conjugated (1:10,000; Cytiva NA931-RRID:AB_772210), and then washed in TBST. Signal was detected using Amersham ECL Western Blotting Detection Reagent (Cytiva, RPN2106) or ECL Prime (Cytiva, RPN2232). Blots were quantified using Fiji Image (Schindelin et al., 2012). Quantifications shown are the mean±s.e.m. of blots from three biologically independent experiments.

To detect protein phosphorylation, protein samples were treated with phosphatase λ (NEB, P0753S), according to manufacturer's instructions. Control samples were incubated without phosphatase. Proteins were separated using SuperSep Phos-tag gels (Wako, 195-16391). Before transfer to nitrocellulose membrane, the gels were washed in transfer buffer containing 10 mM EDTA with a final wash in transfer buffer without EDTA. Immunoblotting was performed as described previously.

To test protein stability, cycloheximide (Abcam, ab120093) was included in the tissue culture media (10 µg/ml) and protein samples subsequently collected at specific timepoints. Protein lysates were then processed as described above.

### RNA sequencing

RNA sequencing was performed on three different biological replicates. RNA was purified from cells using the RNeasy mini kit (Qiagen, 74106) and contaminating DNA removed using the DNA-free DNA removal kit (Invitrogen, AM1906). Poly(A) RNA was selected using NEBNext Poly(A) mRNA Magnetic Isolation Module (E7490) and libraries prepared using the NEBNext Ultra II Directional RNA Library Prep Kit for Illumina (E7760) and NEBNext Multiplex Oligos for Illumina (E6440), following manufacturer's instructions. Samples were sequenced as paired-end 50 bp reads using the NovaSeq 6000 system (Illumina).

### RNA-seq analysis

Adapter and quality trimming were conducted using fastp (Chen et al., 2018). Reads were aligned to the mouse genome version mm39 using STAR (Dobin et al., 2013), then sorted and indexed with samtools (Li et al., 2009). Read counts per gene were obtained using featureCounts (Liao et al., 2014). Differential expression analysis was conducted using DESeq2 (Love et al., 2014), using lfcShrink and apeglm for the shrinkage estimator. Gene ontology analysis was conducted using enrichGO (Wu et al., 2021) and rrvgo (Sayols et al., 2023) used to filter terms based on similarity. The ten most significant terms are shown on each plot.

### Assay for transposase-accessible chromatin (ATAC) sequencing

ATAC-seq was performed on four different biological replicates according to the protocol (Corces et al., 2017). Briefly, 50,000 cells were resuspended in 50 µl of cold resuspension buffer [RSB: 10 mM Tris-HCl (pH 7.4), 10 mM NaCl and 3 mM MgCl_2_] containing 0.1% Tween-20, 0.1% NP40 and 0.01% digitonin. After a 3 min incubation on ice, 300 µl of ice-cold RSB buffer containing 0.1% Tween-20 was added. Nuclei were pelleted and resuspended in 50 µl of transposition mixture (7.5 µl TDE1, 1×TD, 0.01% digitonin, 0.1% Tween-20 in PBS; Qiagen 20034210) and incubated for 30 min at 37°C. The tagmented DNA was purified using DNA Clean up and Concentrator kit (Zymo Research, D4014). ATAC-seq libraries were sequenced as paired-end 100 bp reads using the NovaSeq 6000 system (Illumina).

### ATAC-seq analysis

Adapter and quality trimming were conducted using fastp (Chen et al., 2018). Reads were aligned to the mouse genome version mm39 using bowtie2 (Langmead and Salzberg, 2012). Reads were filtered for properly paired, uniquely mapping reads and fragment size less than 135 bp (a nucleosome free region width) with sambamba (Tarasov et al., 2015). For coverage plots, deepTools (Ramírez et al., 2016) bamCoverage was used to generate bigwig files with data normalised as reads per genomic content, and the average across replicates was obtained using WiggleTools. Peaks were called using MACS2. Differentially accessible regions were determined using DiffBind. Genes proximal to ATAC peaks were assigned using ChIPseeker (Yu et al., 2015) and gene ontology analysis conducted on these proximal genes using enrichGO (Wu et al., 2021), with rrvgo (Sayols et al., 2023) used to filter terms based on similarity. *De novo* motif analysis was conducted using HOMER. The five most significant motifs are shown and transcription factor families with motifs most similar to these are indicated.

## Supplementary Material

10.1242/develop.204329_sup1 Supplementary information
