# Supplementary material for: Phospho-regulation of ASCL1-mediated chromatin opening during cellular reprogramming
Source: Development. 2024 Dec 12;151(24):dev204329. doi: 10.1242/dev.204329 (PMC11664170; doi:10.1242/dev.204329)
Supplement: Supplementary information [file develop-151-204329-s1.pdf]

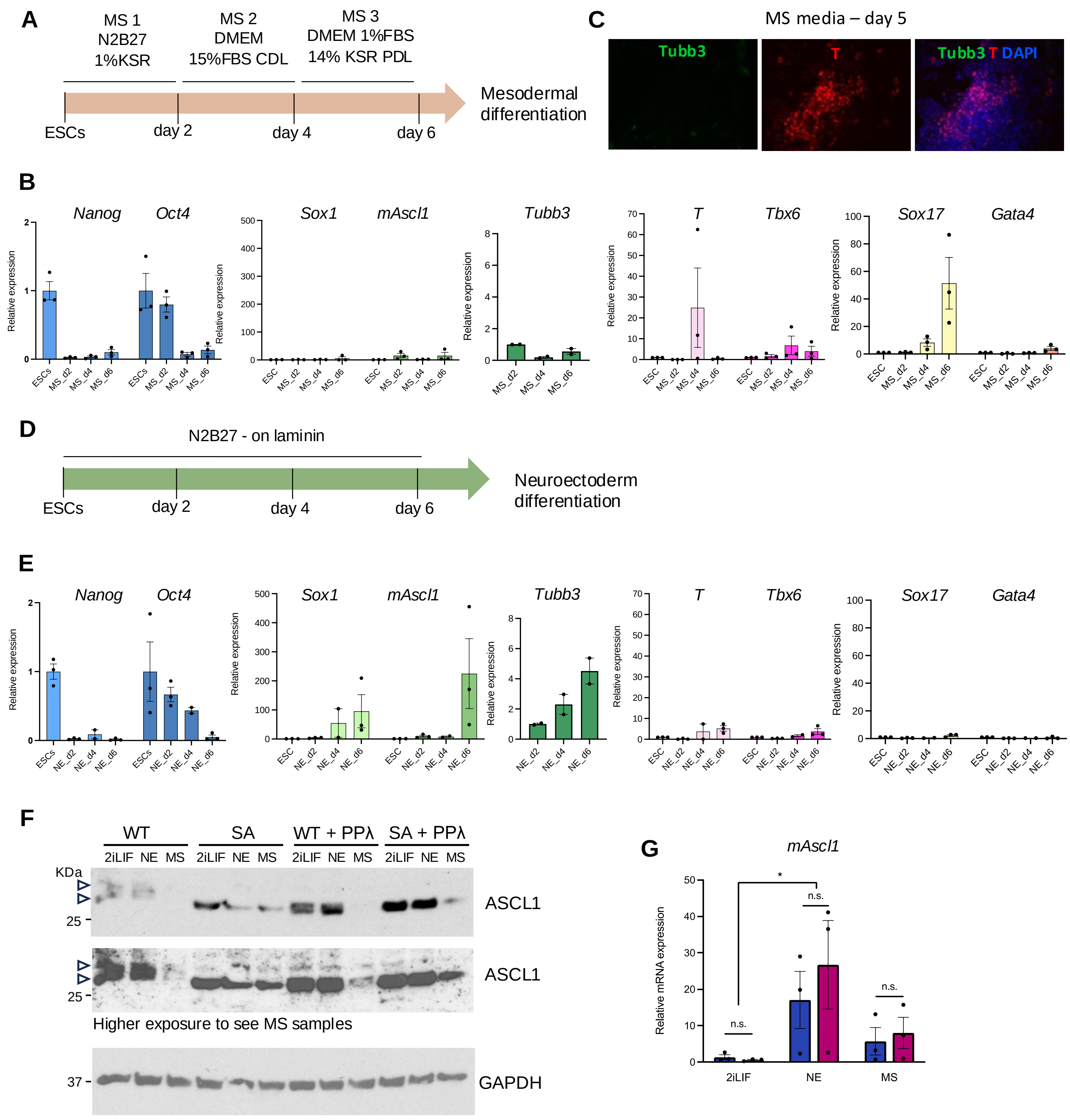


**Fig. S1. Characterization of the differentiation protocols and of ectopic and endogenous ASCL1 expression.** (A and D) Timelines of the differentiation protocols based on Chal et al. 2018 (MS) and Mulas et al. 2019 (NE). KSR: knockout serum, CDL: Chiron-DMSO-LDN193189 (BMP inhibitor), PDL: PD173074 (FGF inhibitor)-DMSO-LDN193189. (B and E) Relative mRNA expression from qPCR of pluripotency, neural and mesodermal genes during MS (B) and NE differentiation (E). Mean +/- s.e.m. (n=3 biological replicates). (C) Immunochemistry showing T (brachyury) expression after 5 days of MS differentiation. (F) Western blots showing exogenous WT and SA ASCL1 protein expression and phosphorylation in pluripotency (2iLIF), neuroectoderm (NE) or mesoderm (MS). White arrowheads indicate the phosphorylated forms of ASCL1 running at higher levels than SA ASCL1 or WT ASCL1 treated with the lambda phosphatase (PPλ). (G) Endogenous *mAscl1* mRNA in pluripotency (2iLIF), neuroectoderm (NE) and mesoderm (MS). Mean +/- s.e.m. (n=3 biological replicates). Unpaired student's t-test between WT and SA in each media. A Two-way ANOVA followed by Tukey's post-hoc test to test the effect of the media. *<p0.01. Each value is mean +/- s.e.m. (n=3 biological replicates).

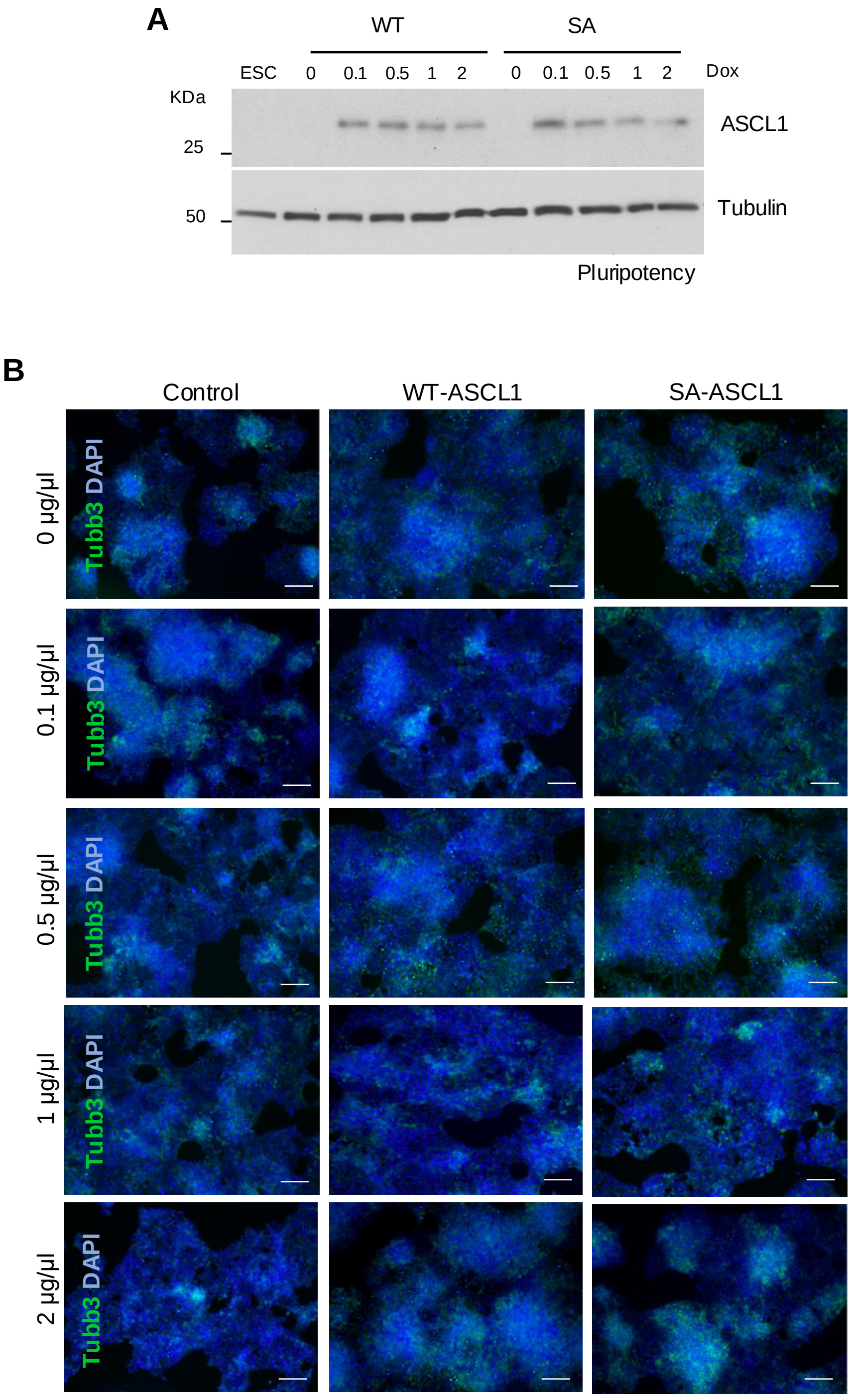


**Fig. S2. Effect of increasing doxycycline levels to induce exogenous ASCL1 in pluripotency.** (A) Western blots showing WT and SA ASCL1 protein levels induced by increasing dox concentrations (in µg/ml), in pluripotent 2iLIF conditions; untreated ESCs are shown as a control. (B) Representative immunostaining images for the neuronal marker Tubb3 (green) in pluripotent cells after 48h of WT ASCL1 or SA ASCL1 overexpression or in control cells using increasing dox concentrations. DAPI nuclear counterstain (blue). Scale bars=100µm.

Development • Supplementary information

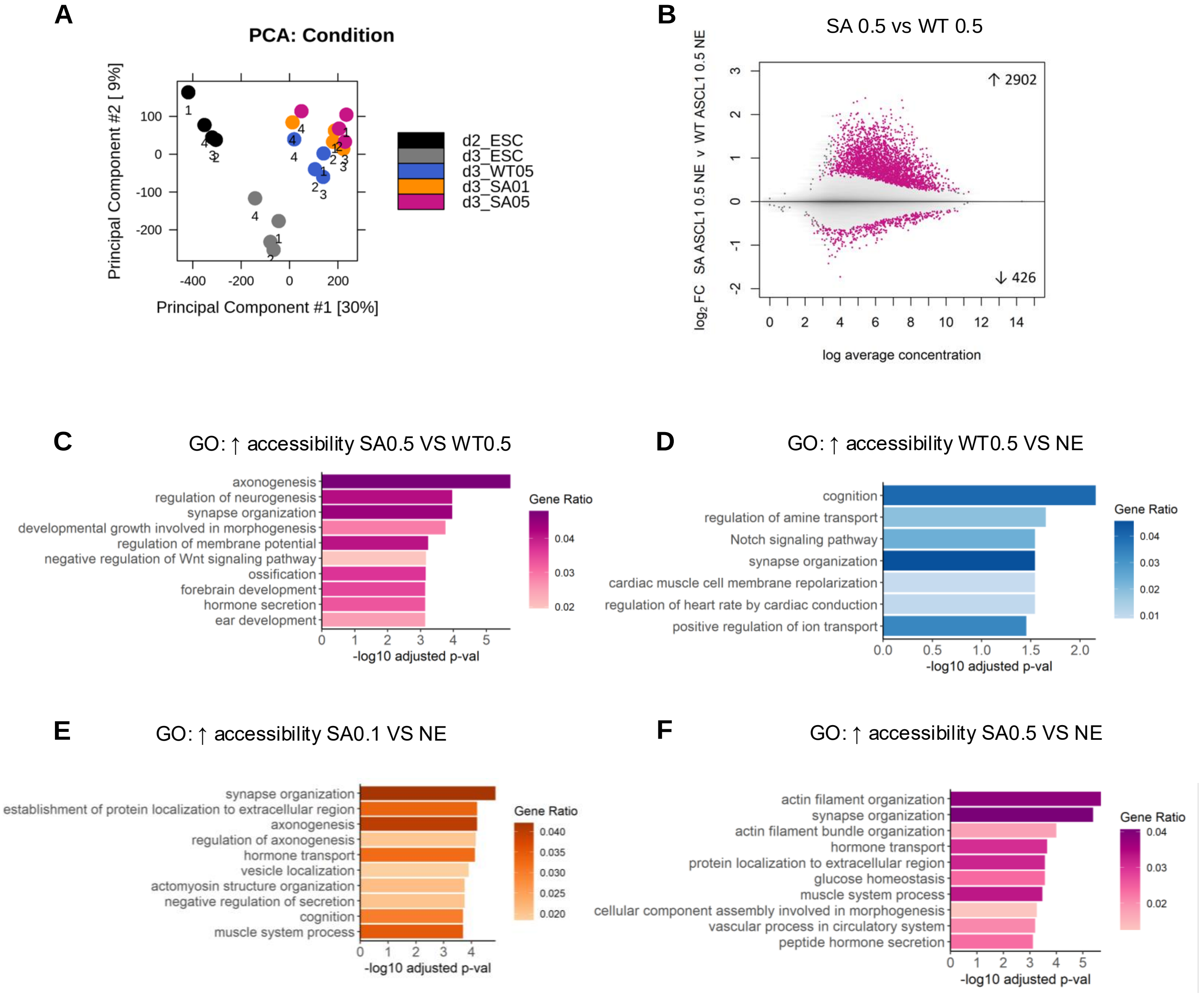


**Fig. S3. ATAC-seq analysis in neuroectoderm.** (A) Principal component analysis (PCA) of ATAC-seq data from the following samples: control cells on day two (d2_ESC) and day three (d3_ESC) of neuroectoderm differentiation; and cells differentiated down the neuroectoderm lineage for three days with a 24hour induction of WT ASCL1 using doxycycline at 0.5 µg/ml (d3_WT0.5) or 24hour induction of SA ASCL1 using doxycycline at 0.1 µg/ml (d3_SA0.1) or 0.5 µg/ml (d3_SA0.5). (B) Graph showing differentially accessible regions (DARs) between neuroectoderm cells after induction of SA ASCL1 (SA) or WT ASCL1 (WT) using dox treatment at 0.5 µg/ml (0.5) for 24hours. (C-F) Gene Ontology (GO) analysis for biological processes on the genes associated with increased accessibility in SA 0.5 versus WT 0.5 (C), in WT 0.5 versus control NE (D), in SA 0.1 versus control NE (E) and in SA 0.5 versus control NE (F).

Development • Supplementary information

**A**

WT 0.5 vs NE

| *de novo* motif | log p-value | % of targets | % of background | similar binding motifs |
|---|---|---|---|---|
| 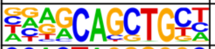 | -1.534e+03 | 32.43 | 13.28 | bHLH family (ASCL1) |
| 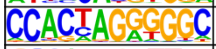 | -1.136e+03 | 17.77 | 5.70 | BORIS, CTCF |
| 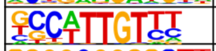 | -3.539e+02 | 22.18 | 13.52 | Sox family |
| 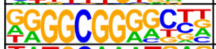 | -2.681e+02 | 15.60 | 9.17 | Sp family, KLF family |
| 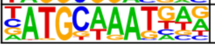 | -2.463e+02 | 4.43 | 1.50 | POU family |

**B**

SA 0.1 vs NE

| *de novo* motif | log p-value | % of targets | % of background | similar binding motifs |
|---|---|---|---|---|
| 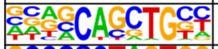 | -4.329e+03 | 51.09 | 21.48 | bHLH family (ASCL1) |
| 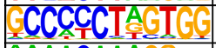 | -1.488e+03 | 12.21 | 3.39 | BORIS, CTCF |
| 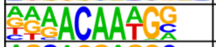 | -4.714e+02 | 18.40 | 11.14 | Sox family |
| 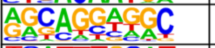 | -4.647e+02 | 18.61 | 11.35 | ~CTCFL ~bHLH family |
| 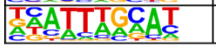 | -2.973e+02 | 5.08 | 2.19 | POU family |

**C**

SA 0.5 vs NE

| *de novo* motif | log p-value | % of targets | % of background | similar binding motifs |
|---|---|---|---|---|
| 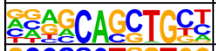 | -4.662e+03 | 46.70 | 19.25 | bHLH family (ASCL1) |
| 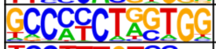 | -1.929e+03 | 17.91 | 6.25 | BORIS, CTCF |
| 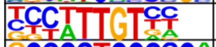 | -6.586e+02 | 23.87 | 14.99 | Sox family |
| 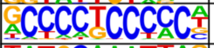 | -5.388e+02 | 10.51 | 5.23 | ZNF148, MAZ |
| 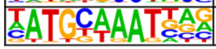 | -3.686e+02 | 6.42 | 3.02 | POU family |

**D**

SA 0.5 vs WT 0.5

| *de novo* motif | log p-value | % of targets | % of background | similar binding motifs |
|---|---|---|---|---|
| 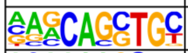 | -2.430e+03 | 83.51 | 22.63 | bHLH family (ASCL1) |
| 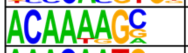 | -1.103e+02 | 21.04 | 11.52 | Sox family |
| 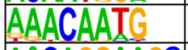 | -1.031e+02 | 18.90 | 10.16 | Sox family |
| 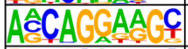 | -8.793e+01 | 22.25 | 13.38 | IKZF1, ETS family |
| 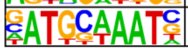 | -8.080e+01 | 11.16 | 5.27 | POU family |

**Fig. S4. Motif analysis of the regions with increased differential accessibility identified in the ATAC-seq analysis in neuroectoderm** (A-D) Tables showing the 5 most significant *de novo* motifs detected using HOMER in the regions with increased accessibility between the conditions indicated.

Development • Supplementary information

**A** RNAseq analysis: Number of genes differentially expressed

| comparison | ↑ | ↓ |
|---|---|---|
| WT ASCL1 0.5 v NE | 2010 | 1118 |
| SA ASCL1 0.1 v NE | 2456 | 1594 |
| SA ASCL1 0.5 v NE | 2728 | 2022 |
| SA ASCL1 0.1 v WT ASCL1 0.5 | 28 | 2 |
| SA ASCL1 0.5 v WT ASCL1 0.5 | 88 | 15 |
| SA ASCL1 0.5 v SA ASCL1 0.1 | 1 | 0 |

**B**

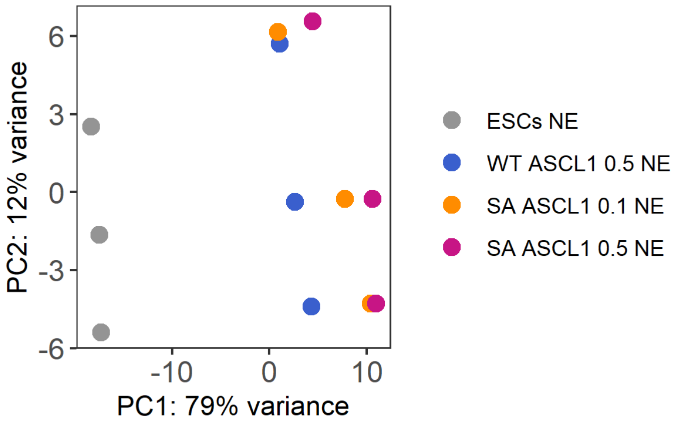


**C**

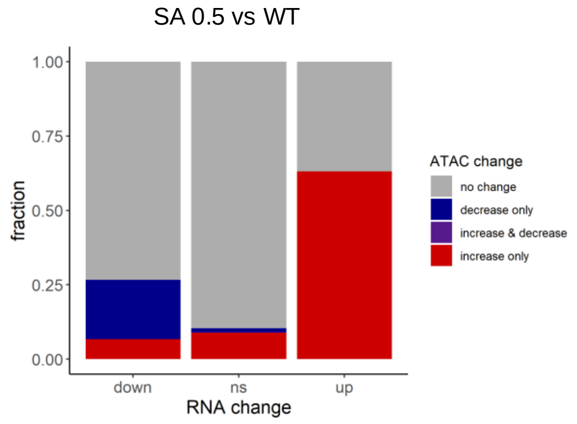


**Fig. S5. RNA-seq analysis in neuroectoderm.** (A) Table showing the number of differentially expressed genes, upregulated (green arrow) and downregulated (red arrow) between conditions; based on p.adj< 0.05 and log2FC > 0.25 or < -0.25. (B) Principal component analysis (PCA) plot of RNA-seq samples of ESCs after 3 days in neuroectoderm media with 24 hours of either WT or SA ASCL1 overexpression. Three biological replicates for each condition. (C) Graph showing correlation between RNAseq and ATACseq data in the SA 0.5 versus WT ASLC1 0.5 comparison. All ATAC peaks were assigned to the most proximal gene as the probable regulatory target. The stacked bar plots show genes grouped by the direction of change (or no significant change) in the RNA-seq and the proportion of these genes that have associated ATAC peaks that increase, decrease or have no change in accessibility.

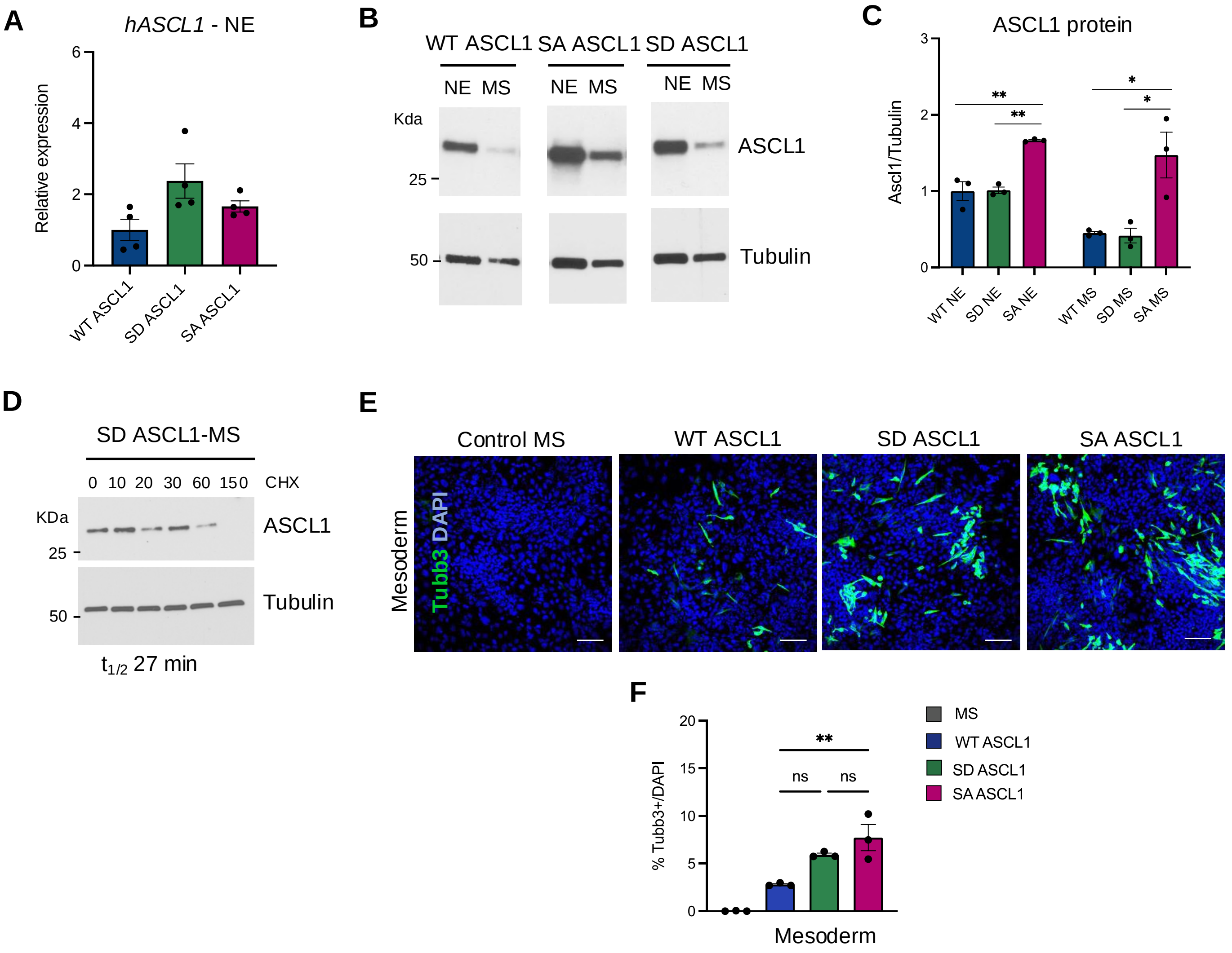


**Fig. S6. Analysis of phosphomimetic ASCL1.** (A-C) Characterization of the cell line overexpressing SD (serine-to-aspartate) ASCL1 mutant. (A) Quantification of ectopic *ASCL1* mRNA normalised to *β-actin* in neuroectoderm (NE) after 24hr induction of WT, SD and SA ASCL1. (B) SD ASCL1 protein expression, in comparison to WT and SA ASCL1 in neuroectoderm (NE) and mesoderm (MS) after 24hr of induction. (C) Quantification of ectopic ASCL1 protein relative to α-tubulin. Mean +/- s.e.m. (n=3 biological experiments). One way ANOVA followed by Tukey post-hoc test; *p<0.05, **p<0.01. (D) Representative Western blot used to calculate SD ASCL1 protein stability in mesoderm (MS). Treatment time with cycloheximide (CHX) is indicated in minutes. Protein half-life (t1/2) is the mean of 3 biologically independent experiments, calculated using first order kinetics and simple linear regression. (E) Representative immunostaining images for Tubb3 (green) in mesoderm after 48hours of WT, SD or SA ASCL1 (0.5 µg/ml dox) or in control cells. DAPI nuclear counterstain (blue). Scale bars=100 µm. (F) Quantification of Tubb3 positive cells over the total DAPI positive cells in mesoderm. Mean +/- s.e.m (n=3) One way ANOVA followed by Tukey post-hoc test; **p<0.01. Statistical differences against control MS not shown (MS vs WT n.s. p=0.084; MS vs SD p=0.001; MS vs SA p=0.0002) .

Development • Supplementary information

**Table S1. Oligonucleotide sequences for SD ASCL1 mutagenesis primers**

| **PCR1 mutagenesis SD ASCL1** | **Primer seq 5'-3'** |
|---|---|
| a667g_g668a_antisense | cctgctcctcggggtcgagcgggtcgtaag |
| a667g_g668a | cttacgacccgctcgaccccgaggagcagg |
| t568g_c569a_g570t_t580g_c581a_antisense | gttggagtagttggggtcgatggtgggatccaggacgcctgcctgg |
| t568g_c569a_g570t_t580g_c581a | ccaggcaggcgtcctggatcccaccatcgaccccaactactccaac |
| **PCR2 mutagenesis SD ASCL1** | **Primer seq 5'-3'** |
| t619g_c620a_g621t_antisense | gagtaggatgagaccggatcgccggccatggagttca |
| t619g_c620a_g621t | tgaactccatggccggcgatccggtctcatcctactc |
| 277g_c278a_g279t_antisense | cgcatcagttcgggatcagacgagcgctgtcgcttg |
| t277g_c278a_g279t | caagcgacagcgctcgtctgatcccgaactgatgcg |

**Table S2. List of primers used for qPCRs**

| **Gene** | **Primer seq 5' to 3'** |
|---|---|
| mActinB_FW | GGCTGTATTCCCCTCCATCG |
| mActinB_RV | CCAGTTGGTAACAATGCCATGT |
| hAscl1_FW | CTCAACTTCAGCGGCTTTG |
| hAscl1_RV | CTCATCTTCTTGTTGGCCGC |
| mNanog_FW | TCTTCCTGGTCCCCACAGTTT |
| mNanog_RV | GCAAGAATAGTTCTCGGGATGAA |
| mOct4_FW | CGGAAGAGAAAGCGAACTAGC |
| mOct4_RV | ATTGGCGATGTGAGTGATCTG |
| mSox1_FW | CCCATGCACCGCTACGACA |
| mSox1_RV | GTAGCCCTGAGAGTTGGAGATG |
| mAscl1-FW | ACTTGAACTCTATGGCGGGTT |
| mAscl1-RV | CCAGTTGGTAAAGTCCAGCAG |
| mTubb3_FW | TCCGCCTGCCTTTTCGTCT |
| mTubb3_RV | CCAGTTGTTGCCAGCACCAC |
| mTBra_FW | GCTTCAAGGAGCTAACTAACGAG |
| mTBra_RV | CCAGCAAGAAAGAGTACATGGC |
| mTbx6_FW | GATCGCAGCCAATCCCTTTG |
| mTbx6_RV | TTTCCTCTTCACACGGGCAT |
| mSox17_FW | CACAACGCAGAGCTAAGCAA |
| mSox17_RV | CGCTTCTCTGCCAAGGTC |
| mGata4_FW | CCCCTCATTAAGCCTCAGCG |
| mGata4_RV | GTGGTGGTAGTCTGGCAGTT |
| mMap2_FW | AAGTGGTGACTTGGCTCAGG |
| mMap2_RV | CGTTTCTCTGGGCTCTTGCT |

Development • Supplementary information
